# Expanding the Genotypic Landscape of Congenital Stationary Night Blindness in an Ethnically Diverse Canadian Population

**DOI:** 10.1155/humu/6564149

**Published:** 2026-05-14

**Authors:** Jennifer Ling, Mustansir Pindwarawala, Cheryl Y. Gregory-Evans, Mathew Kirby, Kevin Gregory-Evans

**Affiliations:** ^1^ Department of Ophthalmology and Visual Sciences, University of British Columbia, Vancouver, Canada, ubc.ca; ^2^ Faculty of Medicine, University of British Columbia, Vancouver, Canada, ubc.ca; ^3^ Department of Ophthalmology, BC Children′s Hospital, Vancouver, Canada

## Abstract

Congenital stationary night blindness (CSNB) is a rare and typically nonprogressive group of genetically heterogeneous disorders resulting in impaired night vision and high myopia with varying levels of visual impairment. Despite being a rare disease with a prevalence of 1:294,000, variants in 22 genes have been associated with specific CSNB phenotypes. Approximately, 13% of cases remain without a genetic diagnosis, highlighting the importance of ongoing genetic studies. Clinical and diagnostic information was collected retrospectively from patients who were diagnosed with CSNB. Patients underwent full ophthalmic examination, including best‐corrected visual acuity (BCVA), direct and indirect ophthalmoscopy, retinal imaging, and full field electroretinography. All patients underwent panel‐based genetic testing for complete and incomplete CSNB genes to identify DNA variants from buccal swab samples. In the 49‐patient cohort (from 38 families) with complete and incomplete CSNB, a conclusive molecular diagnosis was found in 30 patients (61.2%) with a known disease‐causing variant in a CSNB gene. We identified 21 novel variants in five genes (*CACNA1F*, *NYX*, *TRPM1*, *GPR*197, and *GRK1*), and in one patient, the genetic defect remains to be identified. After *in silico* modeling and clinical correlation, 18 of these novel variants were considered pathogenic or likely pathogenic. When compared with ethnicity, CSNB was overwhelmingly present in White people, and we did not find any Indigenous people with CSNB. Prevalence of CSNB in the Mennonite community was estimated to be 1:967, approximately 300 times the expected prevalence. Establishing a molecular diagnosis of CSNB is critical because it enables many actionable outcomes including further family testing, genetic counseling, and access to future clinical trials.

## 1. Introduction

Congenital stationary night blindness (CSNB) with a prevalence of 1:294,000 [1] is a group of inherited retinal diseases (IRDs) characterized by nonprogressive night blindness (nyctalopia) from birth or early life, and often associated with subnormal visual acuity, photophobia, refractive error, nystagmus, and/or strabismus [2]. There are four subtypes of CSNB, where two diseases are associated with a relatively normal‐appearing fundus (Riggs‐type CSNB [3] and Schubert–Bornschein–type CSNB [4]) and two with an abnormal‐appearing fundus (Oguchi disease [5] and fundus albipunctatus [6]), which that both have a Riggs‐type electroretinogram (ERG) pattern. Oguchi disease has a characteristic golden yellow fundus, which becomes normal colored after prolonged dark adaptation, whereas fundus albipunctatus has white–yellow dots in the perimacular and/or peripheral retina.

In addition to clinical findings, each disease has characteristic findings on full‐field electroretinogram (ffERG) testing. In Riggs‐type CSNB, rod photoreceptor dysfunction leads to a severely reduced or absent scotopic response affecting both a‐ and b‐wave responses, whereas photopic responses are largely normal. The Schubert–Bornschein–type can be further divided into incomplete congenital stationary night blindness (icCSNB) and complete congenital stationary night blindness (cCSNB). Though both have relatively normal a‐waves in scotopic and photopic conditions, the b‐wave is significantly reduced under light‐adapted (LA) 3.0 cd‐s‐m^−2^ conditions in the icCSNB form while the b‐wave has a sharply rising peak in the cCSNB form. The LA 30 Hz flicker response is also significantly reduced in icCSNB, but generally preserved in cCSNB, although a delay in the period of the flicker is a well‐known characteristic [7]. In Oguchi disease, which has a Riggs‐type ERG, there is a reduction in a‐wave amplitudes and almost complete absence of b‐waves with flicker responses remaining intact [8]. However, with prolonged dark adaptation, the ERG response to a single flash has normal a‐ and b‐wave amplitudes [9]. In fundus albipunctatus, which also has a Riggs‐type ERG, under scotopic conditions the response to a dim flash is undetectable or shows severe reduction, whereas a bright flash shows a reduced a‐wave. Photopic ERGs are mildly abnormal and often show a flicker delay. Similar to Oguchi disease, prolonged dark adaptation often results in significant recovery of rod‐mediated ERG amplitudes but can be variable [10].

Twenty‐two CSNB genes and more than 360 disease‐causing variants have been identified demonstrating extensive genetic heterogeneity [11–36]; however, genotype–phenotype correlation has associated individual genes with specific subtypes of CSNB. Variants in genes involved in rod photoreceptor function are implicated in Riggs‐type CSNB, Oguchi disease, and fundus albipunctatus, whereas mutations in genes involved in photoreceptor–bipolar signal transduction are implicated in incomplete and complete CSNB. Riggs‐type CSNB is inherited in an autosomal dominant (AD) manner in association with three genes (*GNAT1*, *PDE6B*, and *RHO*) or autosomal recessive (AR) pattern associated with the *GNAT1* and *SLC24A1* genes. In addition, biallelic variants in the *GUCY2D* gene are associated with a Riggs‐like phenotype [33]. Oguchi disease is an AR disease caused by variants in the *SAG* or *GRK1* genes. Fundus albipunctatus is associated with AR variants in *RDH5*, *RLBP1*, and *RPE65*. Schubert–Bornschein–type CSNB occurs in either an X‐linked (XL) or AR pattern. *CACNA1F* variants are associated with XL‐icCSNB, whereas *CABP4*, *CACNA2D4*, and *RIMS2* variants are associated with AR‐icCSNB. Similarly, XL‐cCSNB is seen with variants in *NYX*, whereas AR‐cCSNB is associated with *GRM6*, *TRPM1*, *GPR179*, and *LRIT3* genes. Biallelic variants in *GNB3* have an unusual CSNB phenotype but would best fit into the complete form of CSNB [34]. Recently, a new AR cCSNB gene has been identified (*EGFLAM*) in families of Moroccan ancestry with a Schubert–Bornschein ERG phenotype [36]. The *EGFLAM* gene encodes Pikachurin, which forms a synaptic connection between the photoreceptor terminal and GRP179 on the ON‐bipolar cell membrane.

The Province of British Columbia (BC) on the western coast of Canada is home to diverse ethnic groups, with significant minority populations of Indigenous peoples, as well as East and South Asians, that show differences in the genotype and phenotype of their IRDs [37, 38]. Here, we present data of a genetic screen in BC for CSNB phenotypes in a broad range of ethnicities.

## 2. Materials and Methods

### 2.1. Study Design and Participants

This is a retrospective cohort analysis over a 10‐year period reviewing the clinical charts of patients with the clinical diagnosis of CSNB who underwent genetic testing in an IRD speciality clinic from 2015 to 2025. Following institutional review board approval (University of British Columbia Clinical Research Ethics Board), a systematic search query of CSNB in the Vancouver Eye Research Database (VERD) consisting of all patients diagnosed with an IRD. This study adhered to the tenets of the Declaration of Helsinki, and participants included in the study provided written informed consent.

### 2.2. Clinical Assessment

CSNB was diagnosed based on clinical findings, including patient and family history, clinical examination, and diagnostic investigations. Best‐corrected visual acuity (BCVA) was expressed in LogMAR units. Multimodal retinal imaging included color fundus photography (wide‐field camera; Optos Inc., Marlborough, Massachusetts) and/or fundus autofluorescence. ffERG, according to ISCEV standards [39], was carried out to confirm the type of CSNB where available. Ethnicity was self‐reported and categorized as White, East Asian (China, Korea, Japan, Malaysia, and the Philippines), South Asian (India, Pakistan, and the Middle East), Indigenous people (including Mexican, First Nations, and Métis), and African.

### 2.3. Molecular Genetic Testing

Patients underwent panel‐based genetic testing from a buccal swab. These samples were screened using either the Blueprint Retinal Dystrophy Panel containing 351 genes (Espoo, Finland) or the Invitae Inherited Retinal Disorders Panel containing 330 genes (San Francisco, United States). Previously reported variants were identified from searches of genetic variation databases (Human Gene Mutation Database: [HGMDhomepage]; and ClinVar: [ClinVar]) and reports from the scientific literature. Variants not identified by these searches then underwent in silico analysis using three tools: SIFT (https://sift.jcvi.org/www/SIFT_enst_submit.html), MutationTaster (https://www.mutationtaster.org/), and PolyPhen‐2 (https://genetics.bwh.harvard.edu/pph2/). The variants identified were interpreted based on the criteria established by the American College of Medical Genetics and Genomics [40, 41]. In addition, the Franklin Genoox platform was used to predict pathogenicity for variants of uncertain significance (VUS) (https://franklin.genoox.com/clinical-db/home). Segregation of identified variants was carried out in close family members; however, this was not possible in some relatives who either refused the offer of testing in themselves, lived in another country, or were deceased.

## 3. Results

### 3.1. Demographic Results

During the 10‐year study period, we examined a cohort of 49 patients from 38 families comprising 36 affected males (73.5%), 7 affected females (14.3%), 5 affected female carriers (10.2%), and 1 unaffected female carrier (2.0%). At the time of genetic diagnosis, the age range was 4–72 with a median of 18.5 years. Clinical diagnoses comprised icCSNB (59.2%, *n* = 29), cCSNB (20.3%, *n* = 10), high myopia (8.1%, *n* = 4), Oguchi disease (2.1%, *n* = 1), fundus albipunctatus (2.1%, *n* = 1), cone‐rod dystrophy (6.1%, *n* = 3), and unaffected carriers (2.1%, *n* = 1). The majority of cases had XL inheritance (86.9%), and only six were AR phenotypes (13.1%). With a prevalence of 1:294,000 for CSNB [1] and the population of BC being 5.7 million in 2024 [42], we estimate there would be 19 cases of CSNB, whereas we found 49 cases.

### 3.2. Genotyping

**Figure 1:**
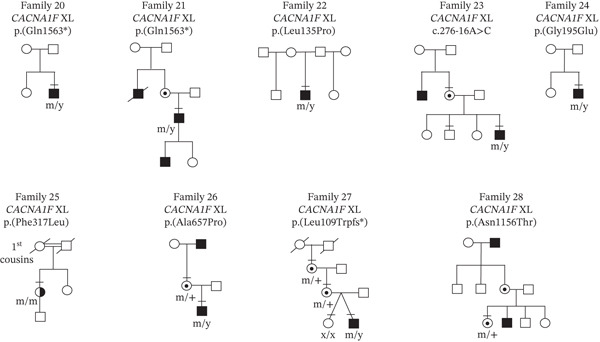
Segregation of novel *CACNA1F* variants in nine families. Squares, males; circles, females; filled symbols, affected; unfilled symbols, unaffected; circles with a central dot, obligate carrier; double line between two people, consanguineous union; bar above symbol, individual examined; m, mutant *CACNA1F* allele; x, normal *CACNA1F* allele; y, Y chromosome; and XL, X‐linked.

**Figure 2:**
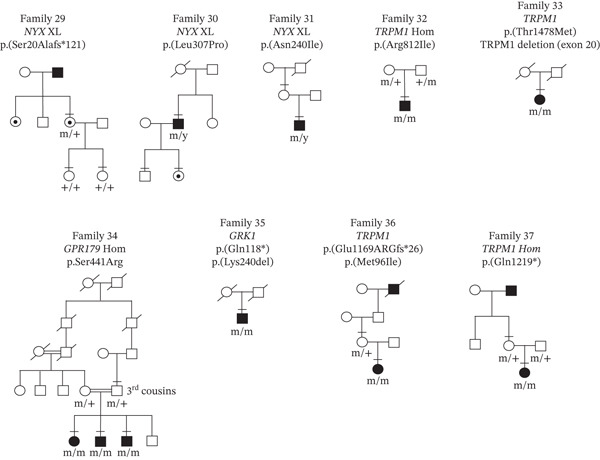
Segregation of novel CSNB gene variants in nine families. Squares, males; circles, females; filled symbols, affected; unfilled symbols, unaffected; circles with a central dot, obligate carrier; double line between two people, consanguineous union; bar above symbol, individual examined; m, mutant allele; +, normal allele; y, Y chromosome; XL, X‐linked; Hom, homozygous variant; and diagonal line, family member is dead.

**Figure 3:**
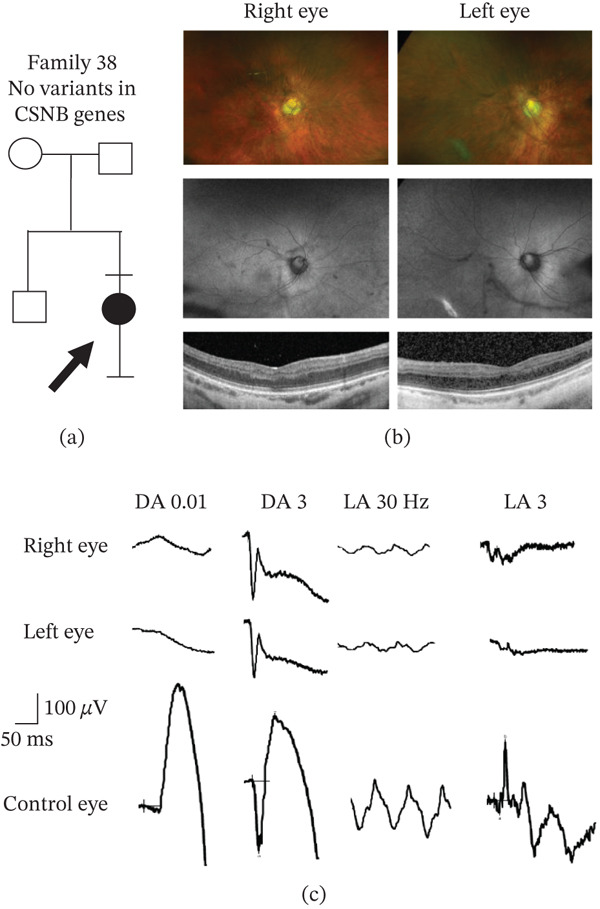
Phenotypic data in a patient from Family 38 with no variants in the known CSNB genes. (a) Family tree. Affected patient (filled‐in circle); brother and parents unaffected (unfilled shapes). Bar above symbol denotes individual examined; (b) Top row, wide‐field fundus images; middle row, fundus autofluorescence images; and bottom row, OCT imaging. (c) Dark‐adapted (DA) and light‐adapted (LA) electroretinogram traces using ISCEV standard flashes consistent with icCSNB.

In the 49‐patient cohort, genetic testing revealed a total of 27 independent CSNB gene variants with one variant [*CACNA1F* c.3166dup, p.(Leu1056Profs ^∗^11)] occurring 24 times (Table S1), with segregation through 14 families (Figure S1). These independent variants comprised 22.2% (6/27) frameshifts, 7.4% (2/27) deletion, 51.9% (14/27) missense, 11.1% (3/27) nonsense, and 7.4% (2/27) intronic variants. When correlated with clinical phenotype, there were 30/49 patients (61.2%) who had a conclusive molecular diagnosis with a known, disease‐causing variant in a CSNB gene (segregation of alleles are shown in Figures S1 and S2). In 18/49 patients (36.7%), 21 novel variants were identified in five CSNB genes (Table 1), which comprised 3 nonsense, 4 frameshifts, 2 deletions, 11 missense changes, and 1 splicing variant. Segregation of novel variants in families where available is shown in Figures 1 and 2. The majority of the novel variants (70%) were absent in the Genome Aggregation Database (gnomAD). In 1/49 patients (Family 38), no variants were identified in any of the known CSNB genes (Figure 3a). She had high myopia and longstanding night blindness. Multimodal imaging showed tilted optic discs and foveal hypoplasia (Figure 3b). Electrophysiology in the subject confirmed they had an electronegative ERG (Figure 3c). Although an electronegative ERG can be seen in other genetic diseases (e.g., retinoschisis, choroideremia, and peripherin‐associated retinopathy) and in some nongenetic conditions (e.g., birdshot retinopathy, central retinal vein occlusion, and melanoma‐associated retinopathy) [43], the clinical examination did not match any of these conditions, and all the genetic conditions were included in the initially genetic screen. Therefore, in this patient the negative ERG is most likely explained by a mutation in a novel gene causing icCSNB.

**Table 1:** Novel variants identified in 18 families with CSNB genes variants.

Patient family	Sex	Age	Ethnicity	Disease	Variants	Variant status	Other variants identified
Fam 20	M	12	White	icCSNB	*CACNA1F* c.4687C>T, p.(Gln1563∗)	Pathogenic	None
Fam 21	M	63	White	CRD	*CACNA1F* c.3887del, p.(Arg1296Profs∗41)	Pathogenic	PITPNM3 c.2762A>G, p.(Asn921Ser) VUS, BP4
Fam 22	M	10	White	icCSNB	*CACNA1F* c.404T>C, p.(Leu135Pro)	Likely pathogenic	PROM1 c.2555A>G, p.(Tyr852Cys) VUS, BP4
Fam 23	M	21	White	icCSNB	*CACNA1F* c.276‐16A>C	VUS (LP)	None
Fam 24	M	20	East Asian	High myopia	*CACNA1F* c.584G>A, p.(Gly195Glu)	VUS	PCARE c.407A>G, p.(Glu136Gly) VUS, BP4 PCARE c.3704C>T, p.Pro1235Leu) VUS, BP4
Fam 25	F	63	East Asian	CRD	*CACNA1F* c.951C>A, p.(Phe317Leu) homozygous	VUS (LP)	None
Fam 26	M	9	White	High myopia	*CACNA1F* c.1969G>C, p.(Ala657Pro)	VUS (LP)	None
Fam 27	M	18	White	High myopia	*CACNA1F* c.325del, p.(Leu109Trpfs∗)	VUS (LP)	None
Fam 28	F	58	African	Cone dystr.	*CACNA1F* c.3467A>C, p.(Asn1156Thr)	VUS (LP)	None
Fam 29	F	42	South Asian	High myopia	*NYX* c.57del, p.(Ser20Alafs∗121)	Pathogenic	None
Fam 30	M	54	White	cCSNB	*NYX* c.920T>C, p.(Leu307Pro)	Likely pathogenic	None
Fam 31	M	19	East Asian	cCSNB	*NYX* c.719A>T, p.(Asn240Ile)	VUS (LP)	None
Fam 32	M	11	South Asian	cCSNB	*TRPM1* c.2435G>T, p.(Arg812Ile) hom	VUS (LP)	None
Fam 33	F	63	White	cCSNB	*TRPM1* deletion (whole exon 20) *TRPM1* c.4433C>T p.(Thr1478Met)	VUS (LP) VUS (LP)	None
Fam 34	F	12	South Asian	cCSNB	*GPR179* c.1321A>C, p.(Ser441Arg) homozygous	VUS (LP)	None
Fam 35	F	72	East Asian	Oguchi	*GRK1* c.352C>T, p.(Gln118∗) *GRK1* c.719_721del, p.(Lys240del)	Pathogenic VUS (LP)	None
Fam 36	F	5	South Asian	cCSNB	*TRPM1* c.3505del, p.(Glu1169Argfs∗26) *TRPM1* c.2886G>A, p.(Met962Ile)	Pathogenic Likely pathogenic	
Fam 37	F	6	South Asian	cCSNB	*TRPM1* c.3655C>T, p.(Gln1219∗) homozygous	Likely pathogenic	None

Abbreviations: BP4, benign supporting classification; cCSNB, complete CSNB; CRD, cone‐rod dystrophy; Cone dystr., cone dystrophy; Fam, Family; icCSNB, incomplete CSNB; VUS, variant of uncertain significance; VUS (LP), reclassified VUS to likely pathogenic classification after clinical correlation (gray‐shaded entries).

### 3.3. Clinical Correlation With In Silico Assessment of Novel CSNB Gene Variants

**Figure 4:**
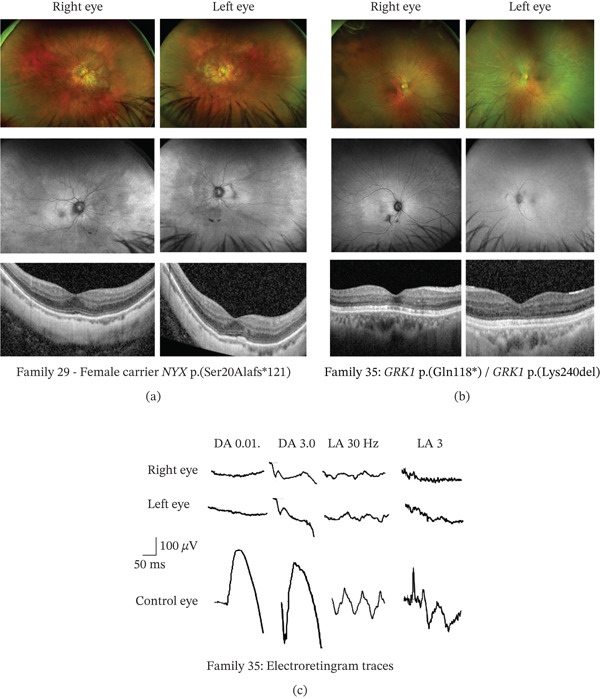
Clinical phenotype in female patients with novel CSNB gene variants. (a) A carrier of an *NYX* variant from Family 29 and (b) a patient from Family 35 with two heterozygous variants in *GRK1*. Top row, wide‐field fundus images; middle row, fundus autofluorescence images; and bottom row, OCT imaging. (c) Dark‐adapted (DA) and light‐adapted (LA) electroretinogram traces using ISCEV standard flashes in the patient from Family 35 consistent with Oguchi disease.

To diagnose the specific type of CSNB in patients, ERG testing is very valuable; however, in some remote parts of BC, this is not possible and in very young children can be unreliable; therefore, clinical findings can help in the diagnosis. The most common clinical finding in patients with novel variants was an abnormal ERG (77.7%, 14/18), followed by abnormal fundus imaging (72.2%, 13/18) and/or high myopia (72.2%, 13/18), nyctalopia (38.9% 7/18), nystagmus (27.7%, 5/18), photophobia (16.7%, 3/18), and strabismus (27.7%, 5/18). In all cases, there were two or more clinical findings supporting a diagnosis of CSNB (Table 2). For example, in Family 29, the affected female had high myopia and nyctalopia and was a carrier of a novel pathogenic *NYX* p.(Ser20Alafs ^∗^121) variant. Her father was also a high myope and night blind and her children were unaffected (Figure 2). An ERG was not available but fundus imaging revealed posterior pole atrophy, and on OCT imaging, the outer retina/RPE was abnormal (Figure 4a). Although rare, carriers of *NYX* variants can show phenotype [44] due to skewed X‐inactivation [45].

**Table 2:** Clinical features in patients with novel CSNB gene variants.

Fam# and sex	Disease	BCVA L:R	Age of onset (years)	Nyctalopia yes/no	High myopia yes/no	Nystagmus yes/no	Photophobia yes/no	Strabismus yes/no	ERG	Imaging
20 M	icCSNB	0.5/0.5	3	Yes	Yes	Yes	No	No	eNEG	Optic disc pallor, depigmented macula, attenuated vessels
21 M	CRD	0.2/0.3	4	No	Yes	No	Yes	No	Abnormal rod/cone responses	Optic disc pallor, macular thinning, color vision loss
22 M	icCSNB	1.0/0.7	7	No	No	Yes	No	No	eNEG	Hearing loss, optic disc pallor
23 M	icCSNB	0.4/0.5	4	No	Yes	No	No	Yes	eNEG	NAD
24 M	High myopia	0.1/0.0	10	No	Yes	No	Yes	No	CRD	Peripheral scarring, schisis, born at 36 weeks
25 F	CRD	0.6/HM	13	No	No	No	Yes	No	N/A	Extensive CRA, consanguineous parents
26 M	High myopia	0.4/0.0	8	No	Yes	No	No	No	N/A	OCT thinning left and right, macular atrophy
27 M	High myopia	0.5/0.7	6	Yes	Yes	Yes	No	Yes	eNEG	Posterior pole atrophy
28 F	Cone dystr.	0.7/0.5	19	No	No	No	No	No	Cone dystrophy	Macular atrophy, pigmentation defects
29 F	High myopia	1.0/1.0	37	Yes	Yes	No	No	No	N/A	Hearing loss
30 M	cCSNB	0.5/0.5	4	Yes	Yes	No	No	No	N/A	Tilted discs, hypo‐autofluorescence
31 M	cCSNB	0.4/0.2	11	No	Yes	Yes	No	Yes	eNEG	Mottled retina, attenuated vessels
32 M	cCSNB	03./0.3	4	No	Yes	No	No	No	eNEG	Tilted discs
33 F	cCSNB	0.0/0.1	10	Yes	No	No	No	No	eNEG	RPE mottling
34 F	cCSNB	0.4/0.8	6	No	Yes	Yes	No	Yes	eNEG	NAD consanguineous parents
35 F	Oguchi	0.5/HM	65	Yes	No	No	No	No	eNEG abnormal rod/cone	Retinal sheen
36 F	cCSNB	0.6/0.54	5	No	Yes	No	No	No	eNEG	NAD
37 F	cCSNB	−0.12/−0.12	6	Yes	Yes	No	No	Yes	eNEG	NAD consanguineous parents

Abbreviations: BCVA, best‐corrected visual in Logmar L: R, left and right; cCSNB, complete CSNB; CRA, chorioretinal atrophy; CRD, cone‐rod dystrophy; Cone dystr., cone dystrophy; eNEG, electronegative electroretinogram; F, female; icCSNB, incomplete CSNB; M, male; MD, macular dystrophy; NAD, nothing abnormal detected; Pt ID, patient identifier; RP, retinitis pigmentosa; RPE, retinal pigment epithelium.

The in silico variant modeling programs predicted that 5 of the 21 novel CSNB variants were pathogenic [*CACNA1F* p.(Gln1563 ^∗^); *CACNA1F* p.(Arg1296Profs ^∗^41); *NYX* p.(Ser20Alafs ^∗^121); *GRK1* c.352C>T, p.(Gln118 ^∗^); and *TRPM1* c.3505del, p.(Glu1169Argfs ^∗^26)] and 4 are likely pathogenic [*CACNA1F* c.404T>C, p.(Leu135Pro); *NYX* c.920T>C, p.(Leu307Pro); *TRPM1* c.2886G>A, p.(Met962Ile); and *TRPM1* c.3655C>T, p.(Gln1219 ^∗^)] as they were predicted to disrupt the protein structure or function. In addition, clinical correlation of these variants showed they were likely disease‐causing (Table 2). For example, in Family 35, an East Asian woman had a pathogenic *GRK1* p.(Gln118 ^∗^) variant and a *GRK1* p.(Lys240del) VUS. Neither of her parents were alive, and she had no children to test for segregation (Figure 2). Fundus imaging revealed a golden sheen, and OCT imaging showed abnormal outer retina (Figure 4b). Electrophysiology suggested she had Oguchi disease (Figure 4c). Therefore, the genetic results confirmed the clinical data.

**Figure 5:**
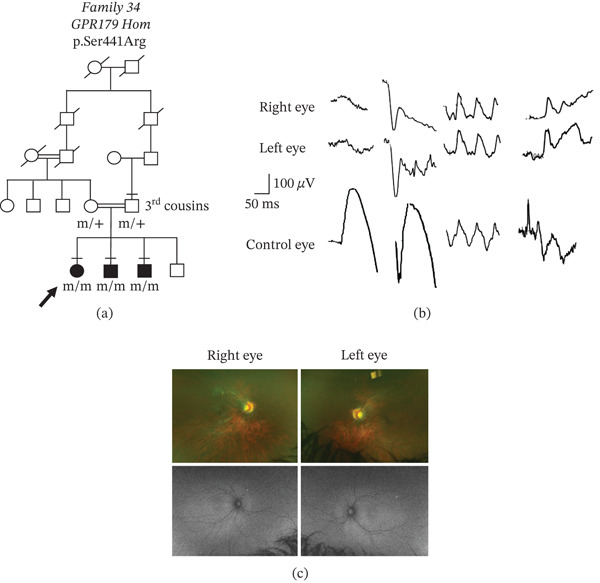
Genetic analysis and phenotypic assessment for a novel homozygous *GPR179* p.Ser441Arg variant. (a) Family tree showing consanguineous union (third cousins) and segregation of the mutant allele in the family. Bar above symbol, individual examined; m, mutant allele; +, normal allele; arrow indicates the individual assessed by electrophysiology and imaging in Parts (b) and (c). (b) Dark‐adapted (DA) and light‐adapted (LA) electroretinogram traces using ISCEV standard flashes consistent with cCSNB. (c) Top row, wide‐field fundus images; Bottom row, fundus autofluorescence images.

**Figure 6:**
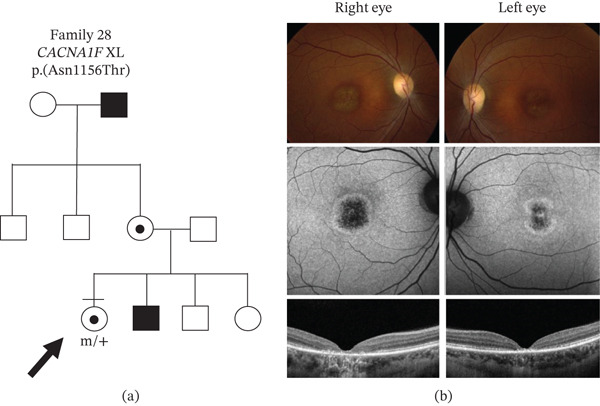
Phenotype of a female carrier of novel *CACNA1F* p.(Asn1156Thr) variant. (a) Family tree showing the patient′s extended family had documented a macular dystrophy phenotype. Bar above symbol, individual examined; m, mutant allele; x, normal X chromosome. Arrow indicates the individual assessed by imaging in Part (b): top row, wide‐field fundus images; middle row, fundus autofluorescence images; and bottom row, OCT imaging.

The remaining 12/21 novel CSNB gene variants were considered VUS. In seven patients (from Families #23, 27, and 31 to 35), there were eight VUS (Table 1), and all patients were confirmed to have CSNB by electrophysiology, had clinical characteristics supporting a CSNB diagnosis (Table 2), and variant segregation was confirmed in 5/7 patients. For example, in Family 34, a 6‐year‐old Algerian girl with high myopia, nystagmus, and strabismus was found to have a homozygous *GRP179* p.(Ser441Arg) VUS. Her two brothers who were also symptomatic were tested and found to carry the same variants. The parents were carriers of the variants and were third cousins, which would explain the homozygous inheritance pattern (Figure 5a). The ERG was electronegative, and her fundus imaging appeared to be normal (Figure 5c). In another patient (Family 28), multimodal imaging of the macular revealed a hyperfluorescent ring surrounding hypofluorescent pigmentation. The outer retina in the OCT imaging showed marked atrophy that supported a diagnosis of cone dystrophy (Figure 6), which was associated with the *CACNA1F* p.(Asn1156Thr) VUS (Table 2). No other variants were detected in this patient. A ffERG showed normal scotopic responses but reduced photopic and flicker responses. The mfERG showed marked reduction of central and paracentral responses. This was consistent with a diagnosis of XL cone dystrophy [46]. *In silico* modeling predicted that 8/9 of these VUS were likely to have consequence for protein function and had not been seen before. The *TRPM1* p.(Thr1478Met) variant in Family 33 was scored by *in silico* programs as benign or not available. However, since the patient had a second variant in *TRPM1* (exon 20 deletion), which was predicted to be deleterious, then the combination of the two *TRPM1* variants is likely to be disease‐causing given the positive clinical correlation. This patient had no children, and her parents were not available for genetic testing to confirm segregation. Finally, no other gene variants were identified in any of these eight patients that could otherwise explain the phenotype. Therefore, these nine VUS were reclassified as likely pathogenic [*CACNA1F* c.276‐16A>C; *CACNA1F* c.325del, p.(Leu109Trpfs ^∗^); *CACNA1F* p.(Asn1156Thr); *NYX* p.(Asn240Ile); *TRPM1* p.(Arg812Ile); *TRPM1* exon 20 del; *TRPM1* p.(Thr1478Met); *GPR179* p.(Ser441Arg); and *GRK1* p.(Lys240del)].

**Figure 7:**
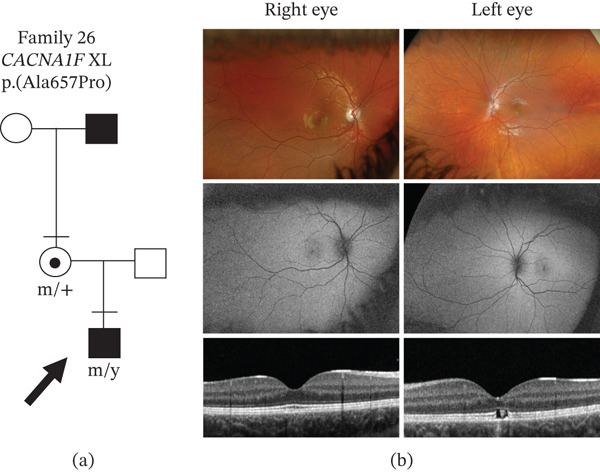
Genetic and clinical phenotype in Family 26 without electrophysiology testing. (a) Family tree showing segregation of the novel *CACNA1F* p.(Ala657Pro) variant. Bar above symbol, individual examined; m, mutant allele; +, normal allele; y, Y chromosome. Arrow indicates the individual assessed by imaging in Part (b): top row, wide‐field fundus images; middle row, fundus autofluorescence images; and bottom row, OCT imaging.

There were two patients with a VUS who had not had ERG testing, but clinical correlation would support reclassifying the VUS as likely pathogenic (Tables 1 and 2). In Family 25, the daughter was carrying a homozygous *CACNA1F* p.(Phe317Leu) VUS, which was predicted to be deleterious by *in silico* modeling. Her parents were first cousins, and she had extensive chorioretinal atrophy and no other gene variants. *CACNA1F* variants have been associated with X‐linked cone‐rod dystrophy (CORDX3) [46]. Some female carriers of *CACNA1F* variants have been reported to have clinical symptoms [47]; thus, this *CACNA1F* p.(Phe317Leu) variant is likely to be disease‐causing. In Family 26, a *CACNA1F* p.(Ala657Pro) VUS was predicted to be deleterious by all in silico programs. At the age of 8 years old, he had high myopia, macular atrophy, and thinning of the retina on OCT imaging (Figure 7). His grandfather was also reported to have macular atrophy. He had no other gene variants that could explain the eye phenotype, suggesting this variant was disease causing.

The final patient (Family 24) with a *CACNA1F* p.(Gly195Glu) VUS remains inconclusive. *In silico* programs predict that this variant is likely to disrupt protein function. At the age of 20 years, he had high myopia and photophobia. Genetic testing revealed two additional variants: [*PCARE* c.3704C>T, p.(Pro1235Leu) and *PCARE* c.407A>G, p.(Glu136Gly)] that were both predicted to be tolerated and are associated with the condition retinitis pigmentosa. ERG testing is needed in this patient to confirm a CSNB diagnosis.

Of the 18 families with 21 novel variants, one family had a Riggs ERG phenotype with confirmed Oguchi disease and fundus abnormalities. The remainder had variants in genes associated with the Schubert–Bornschein ERG phenotype. There were four families confirmed to have icCSNB, and six confirmed with cCSNB by clinical evaluation, genetics, and electrophysiology.

### 3.4. Correlation With Ethnicity

The ethnicity of the patients compared with BC population census statistics [42] denoted in square brackets were 69.4% White (*n* = 34 [BC census 55.3%]), 16.2% South Asian (*n* = 8 [13.5%]), 12.2% East Asian (*n* = 6 [21.6%]), and 2.3% African (*n* = 1 [1.7%]). We did not find any Indigenous people with a CSNB phenotype even though they represent 7.9% of the BC population. We noted an overrepresentation of White people with CSNB and an underrepresentation in East Asian people. The 28 subjects with previously reported variants in CSNB genes were overwhelmingly of White origin (86.7%, 26/30 cases). In the 18 subjects with novel variants in CSNB genes, the ethnic breakdown was 44.4% (8/18 cases) White, 27.8% (5/17 cases) South Asian, 22.2% (4/17 cases) East Asian, and 5.6% (1/18 cases) African. Thus, the majority of novel genes variants were in 55.6% of non‐White subjects. The single subject with no variant in any CSNB gene was of South Asian descent.

## 4. Discussion

Here, we describe a retrospective analysis of the genotypic and phenotypic spectrum of a Western Canadian cohort of patients carrying CSNB gene variants. We identified 49 patients, which is higher than the expected 19 cases, based on the CSNB disease prevalence of 1:294,000 and the 5.7 million population of BC. This higher prevalence of CSNB is most likely explained by the 24 cases who all had the same variant in *CACNA1F* (c.3166dup, p.(Leu1056Profs ^∗^11). After further consultation with these 24 patients, 12 were known to be related to one another. This variant has been previously reported as a founder mutation in people of the Mennonite religious community in Canada [48]. Based on the 2021 Canada census, there were 144,045 Mennonites in Canada in total, and in BC, there were 21,280 [49], who mostly reside in the Fraser Valley where all the patients with the founder variant are from. In this particular region, the majority of residents are part of the farming population. Thus, within this defined geographical location, the prevalence of CSNB is estimated to be 1:967, approximately 300 times the expected prevalence. In other retinal diseases we have studied in BC, such as *RPGR*‐related retinitis pigmentosa [50], *ABCA4*‐related retinopathy [51], and *USH2A*‐related retinitis pigmentosa [37], we find that these diseases match the expected prevalence rates.

A conclusive molecular genetic diagnosis was identified in 61.2% of CSNB cases who were mostly of White ethnicity (86.7%). In the remaining cases we identified 21 novel variants and used *in silico* modeling to determine pathogenicity. This analysis predicted that five were pathogenic and four were likely pathogenic. Furthermore, another nine variants were reclassified from VUS to likely pathogenic, taking into account clinical correlation and electrophysiological testing. Therefore, the conclusive diagnostic rate was increased from 61.2% to 89.8% (44/49 cases), comparable with other studies [2, 52].

Some female carriers of XL diseases show a mild to moderate phenotype depending on the amount of X‐inactivation [44]. In ocular diseases, *RPGR*‐associated retinopathy [53] and ocular albinism [54] commonly have symptomatic female carriers. In this study, we found three female carriers with a CSNB phenotype [Family 25, homozygous *CACNA1F* c.951C>A p.(Phe317Leu); Family 28, *CACNA1F* c.3467A>C, p.(Asn1156Thr), and Family 29, *NYX* c.57del, p.(Ser20Alafs ^∗^121)]. Although rare, carriers have been previously reported for *CACNA1F* [45] and *NYX* variants [44]. This suggests that assessment of X‐inactivation could be important as well as longitudinal studies of the phenotype. Female carriers of CSNB gene variants could benefit from treatment trials and future gene therapies as have the cohort of female carriers of *RPGR* variants in current clinical trials.

Currently, there are no gene therapy trials for any of the CSNB genes; however, based on animal models research, some trials may be on the horizon. Nyctalopin expression in retinal bipolar cells in a mouse model of CSNB (*Nyx^nob^*) rescued ERG b‐wave activity and restored TRPM1 protein localization to bipolar cell dendrites [55]. Similarly, delivery of *Lrit3* to Lrit3 knockout mice restored b‐wave activity to 50% of wildtype and restored expression of TRPM1 [56]. A beagle dog model was recently identified with a mutation in the *LRIT3* gene causing loss of scotopic ERG b‐waves [57]. An AAV‐based delivery strategy for *LRIT3* resulted in improving the ERG b‐wave to 30% of normal levels, which was detectable for up to 32 months after treatment [58]. While successful results have been achieved in animal models for CSNB gene therapy, we await trials to begin in human patients.

## 5. Conclusion

Here, we report the mutation spectrum in a cohort of CSNB patients from Western Canada. A correct diagnosis is particularly relevant for the patients, as it is nonprogressive, unlike other diseases like RP, which would be considered in the differential diagnosis. A key diagnostic test is electroretinography; however, in some remote areas of Canada, this is not available; therefore, CSNB maybe underdiagnosed. In clinics without access to ERG testing, gene variant identification could help in the diagnosis of CSNB. Subsequent follow‐up at specialist clinics could then be leveraged. We identified 21 novel variants in five genes and describe the genotype–phenotype correlation. The prevalence of CSNB in the Mennonite community in Western Canada is estimated to be 1:967 compared with the 1:294,000 expected prevalence. This is very important in terms of genetic counseling and patient management, particularly with respect to future treatment options.

## Author Contributions

J.L.: formal analysis; writing—review & editing; M.P.: formal analysis; writing—review & editing; C.Y.G‐E.: data curation; formal analysis; writing—original draft; writing—review & editing; M.K.: data curation; writing—review & editing; K.G‐E.: conceptualization; funding acquisition; investigation; data curation; formal analysis; writing—review & editing.

## Funding

This study was supported by Fighting Blindness Canada.

## Conflicts of Interest

The authors declare no conflicts of interest.

## Supporting Information

Additional supporting information can be found online in the Supporting Information section.

## Supporting information


**Supporting Information 1** Table S1: Previously reported CSNB gene variants identified in this study.

**Supporting Information 2** Figure S1: Segregation of the *CACNA1F* founder variant c.3166dup, p.(Leu1056Profs ^∗^11) in 14 families. Squares, males; circles, females; diamonds, offspring of unknown sex; filled symbols, affected; unfilled symbols, unaffected; circles with a central dot, obligate carrier; half‐filled symbols, affected carrier; symbols with a number, the number of offspring; bar above symbol, individual examined; m, mutant *CACNA1F* allele; x, normal *CACNA1F* allele; and y, Y chromosome.

**Supporting Information 3** Figure S2: Segregation of previously reported CSNB variants in five families. Squares, males; circles, females; filled symbols, affected; unfilled symbols, unaffected; circles with a central dot, carrier; bar above symbol, individual examined; m, mutant allele; x, normal X allele; y, Y chromosome; and +, wildtype allele.

## Data Availability

The data that support the findings of this study are available on request from the corresponding author. The data are not publicly available due to privacy or ethical restrictions.
