# Supplementary material for: Expanding the Genotypic Landscape of Congenital Stationary Night Blindness in an Ethnically Diverse Canadian Population
Source: Hum Mutat. 2026 May 14;2026:6564149. doi: 10.1155/humu/6564149 (PMC13176619; doi:10.1155/humu/6564149)
Supplement: Supplementary file 1 — Supporting Information 1 Table S1: Previously reported CSNB gene variants identified in this study. [file HUMU-2026-6564149-s001.docx]

**Table S1.** Previously reported CSNB gene variants identified in this study

| **Family #** | **Sex** | **Age** | **Ethnicity** | **CSNB gene variant** | **Disease** |
| --- | --- | --- | --- | --- | --- |
| Fam 1 | F | 66 | E. Asian | *RDH5* c.928delinsGAAG, p.(Leu310delinsGluVal) - hom | FA |
| Fam 2 | M | 19 | White | *CACNA1F* c.325del, p.(Leu109Trpfs*28) | icCSNB |
| Fam 3 | M | 16 | S. Asian | *TRPM1*c.2999G>A, p.(Arg1000Gln) – hom | cCSNB |
| Fam 3 | M | 23 | S. Asian | *TRPM1*c.2999G>A, p.(Arg1000Gln) – hom | cCSNB |
| Fam 4 | M | 61 | White | *CACNA1F* c.3052G>A, p.(Gly1018Arg) | icCSNB |
| Fam 5 | M | 14 | E. Asian | *TRPM1* c.220C>T – hom | cCSNB |
| Fam 6 | M | 9 | White | *CACNA1F* c.3166dup, p.(Leu1056Profs*11) | icCSNB |
| Fam 6 | M | 13 | White | *CACNA1F* c.3166dup, p.(Leu1056Profs*11) | icCSNB |
| Fam 6 | M | 47 | White | *CACNA1F* c.3166dup, p.(Leu1056Profs*11) | icCSNB |
| Fam 7 | M | 10 | White | *CACNA1F* c.3166dup, p.(Leu1056Profs*11) | icCSNB |
| Fam 7 | M | 13 | White | *CACNA1F* c.3166dup, p.(Leu1056Profs*11) | icCSNB |
| Fam 8 | M | 15 | White | *CACNA1F* c.3166dup, p.(Leu1056Profs*11) | icCSNB |
| Fam 9 | M | 4 | White | *CACNA1F* c.3166dup, p.(Leu1056Profs*11) | icCSNB |
| Fam 9 | M | 13 | White | *CACNA1F* c.3166dup, p.(Leu1056Profs*11) | icCSNB |
| Fam 9 | M | 15 | White | *CACNA1F* c.3166dup, p.(Leu1056Profs*11) | icCSNB |
| Fam 9 | M | 24 | White | *CACNA1F* c.3166dup, p.(Leu1056Profs*11) | icCSNB |
| Fam 9 | F | 35 | White | *CACNA1F* c.3166dup, p.(Leu1056Profs*11) | normal |
| Fam 10 | M | 12 | White | *CACNA1F* c.3166dup, p.(Leu1056Profs*11) | icCSNB |
| Fam 11 | M | 4 | White | *CACNA1F* c.3166dup, p.(Leu1056Profs*11) | icCSNB |
| Fam 11 | M | 5 | White | *CACNA1F* c.3166dup, p.(Leu1056Profs*11) | icCSNB |
| Fam 11 | F | 39 | White | *CACNA1F* c.3166dup, p.(Leu1056Profs*11) | icCSNB |
| Fam 12 | M | 22 | White | *CACNA1F* c.3166dup, p.(Leu1056Profs*11) | icCSNB |
| Fam 13 | M | 12 | White | *CACNA1F* c.3166dup, p.(Leu1056Profs*11) | icCSNB |
| Fam 13 | M | 33 | White | *CACNA1F* c.3166dup, p.(Leu1056Profs*11) | icCSNB |
| Fam 14 | F | 14 | White | *CACNA1F* c.3166dup, p.(Leu1056Profs*11) | icCSNB |
| Fam 15 | M | 43 | White | *CACNA1F* c.3166dup, p.(Leu1056Profs*11) | icCSNB |
| Fam 16 | M | 5 | White | *CACNA1F* c.3166dup, p.(Leu1056Profs*11) | icCSNB |
| Fam 17 | M | 41 | White | *CACNA1F* c.3166dup, p.(Leu1056Profs*11) | icCSNB |
| Fam 18 | M | 13 | White | *CACNA1F* c.3166dup, p.(Leu1056Profs*11) | icCSNB |
| Fam 19 | M | 19 | White | *CACNA1F* c.3166dup, p.(Leu1056Profs*11) | icCSNB |

CSNB, congenital stationary night blindness, CSNB; F, female; Fam, family; FA, fundus albipunctatus; hom; homozygous; icCSNB, incomplete CSNB; cCSNB, complete CSNB; E. Asian, East Asian; M, male; S. Asian, South Asian
