# Supplementary material for: Expanding the Genotypic Landscape of Congenital Stationary Night Blindness in an Ethnically Diverse Canadian Population
Source: Hum Mutat. 2026 May 14;2026:6564149. doi: 10.1155/humu/6564149 (PMC13176619; doi:10.1155/humu/6564149)
Supplement: Supplementary file 2 — Supporting Information 2 Figure S1: Segregation of the CACNA1F founder variant c.3166dup, p.(Leu1056Profs ∗11) in 14 families. Squares, males; circles, females; diamonds, offspring of unknown sex; filled symbols, affected; unfilled symbols, unaffected; circles with a central dot, obligate carrier; half‐filled symbols, affected carrier; symbols with a number, the number of offspring; bar above symbol, individual examined; m, mutant CACNA1F allele; x, normal CACNA1F allele; and y, Y chromosome. [file HUMU-2026-6564149-s002.pdf]

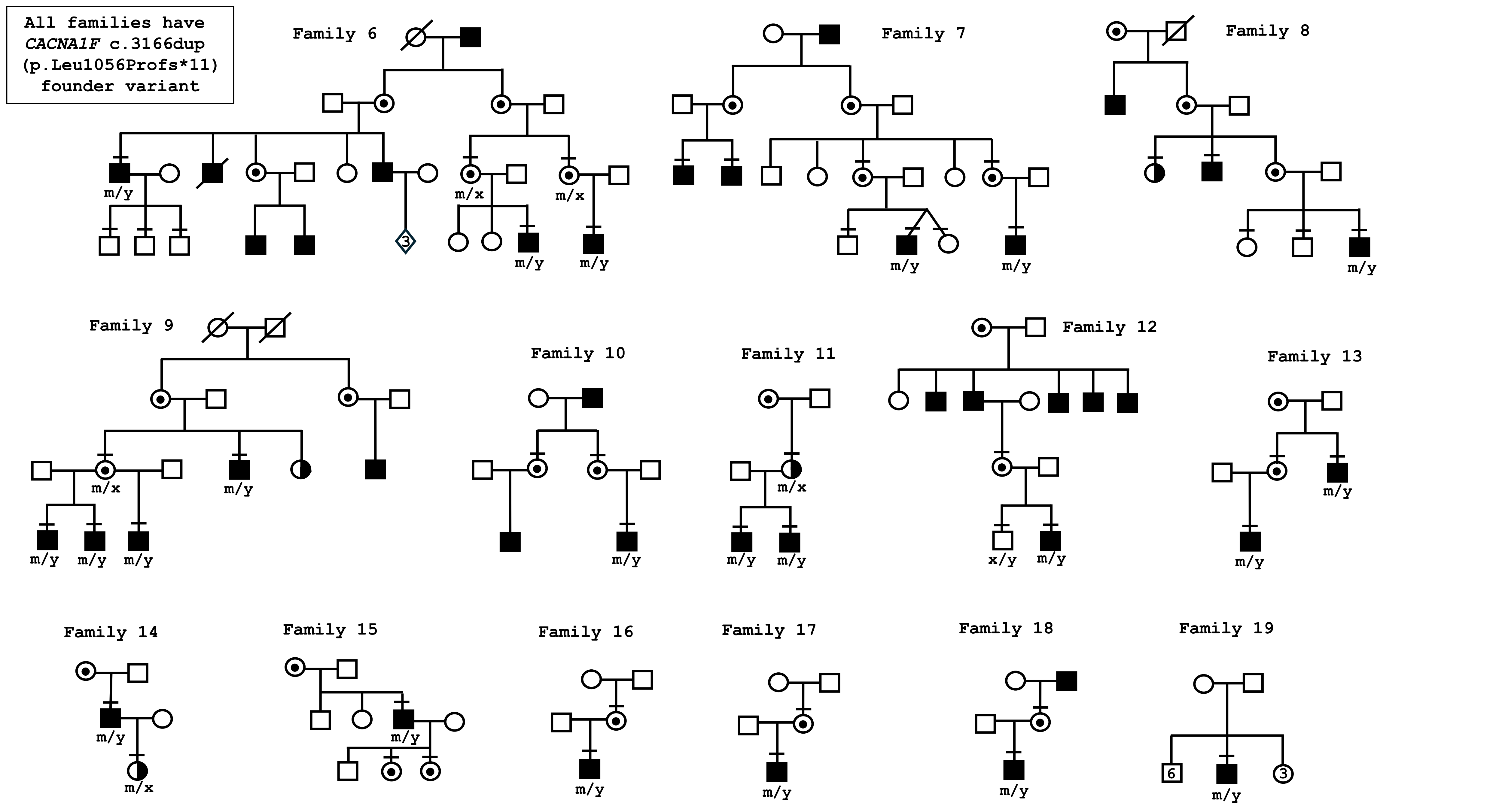
All families have
CACNA1F c.3166dup
(p.Leu1056Profs*11)
founder variant
Family 6
m/y
m/x
m/x
3
m/y
m/y
Family 7
m/y
m/y
Family 8
m/y
Family 9
m/x
m/y
m/y
m/y
m/y
Family 10
m/y
Family 11
m/x
m/y
m/y
Family 12
x/y
m/y
Family 13
m/y
m/y
Family 14
m/y
m/x
Family 15
m/y
Family 16
m/y
Family 17
m/y
Family 18
m/y
Family 19
6
3
m/y
