# Supplementary material for: Expanding the Genotypic Landscape of Congenital Stationary Night Blindness in an Ethnically Diverse Canadian Population
Source: Hum Mutat. 2026 May 14;2026:6564149. doi: 10.1155/humu/6564149 (PMC13176619; doi:10.1155/humu/6564149)
Supplement: Supplementary file 3 — Supporting Information 3 Figure S2: Segregation of previously reported CSNB variants in five families. Squares, males; circles, females; filled symbols, affected; unfilled symbols, unaffected; circles with a central dot, carrier; bar above symbol, individual examined; m, mutant allele; x, normal X allele; y, Y chromosome; and +, wildtype allele. [file HUMU-2026-6564149-s003.pdf]

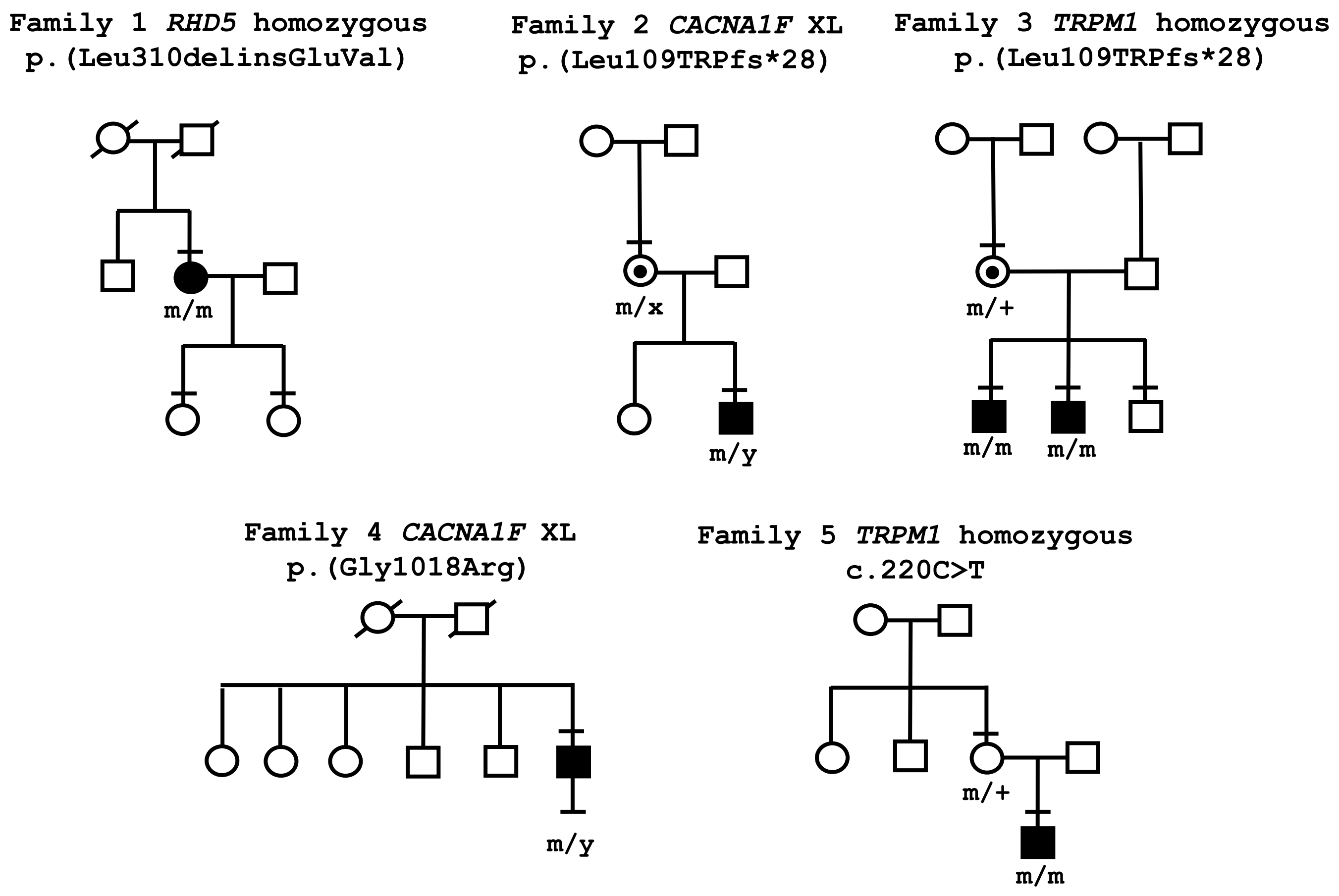

Family 1 *RHD5* homozygous
p.(Leu310delinsGluVal)
m/m
Family 2 *CACNA1F* XL
p.(Leu109TRPfs*28)
m/x
m/y
Family 3 *TRPM1* homozygous
p.(Leu109TRPfs*28)
m/+
m/m
m/m
Family 4 *CACNA1F* XL
p.(Gly1018Arg)
m/y
Family 5 *TRPM1* homozygous
c.220C>T
m/+
m/m
